# A Review of Treatment of Coronavirus Disease 2019 (COVID-19): Therapeutic Repurposing and Unmet Clinical Needs

**DOI:** 10.3389/fphar.2020.584956

**Published:** 2020-11-17

**Authors:** Po-Lin Chen, Nan-Yao Lee, Cong-Tat Cia, Wen-Chien Ko, Po-Ren Hsueh

**Affiliations:** ^1^ Department of Internal Medicine, National Cheng Kung University Hospital, College of Medicine, National Cheng Kung University, Tainan, Taiwan; ^2^ Infection Control Center, National Cheng Kung University Hospital, College of Medicine, National Cheng Kung University, Tainan, Taiwan; ^3^ Department of Microbiology and Immunology, College of Medicine, National Cheng Kung University, Tainan, Taiwan; ^4^ Departments of Laboratory Medicine and Internal Medicine, National Taiwan University Hospital, National Taiwan University College of Medicine, Taipei, Taiwan

**Keywords:** COVID-19, SARS-CoV-2, treatment, anti-viral agents, interleukin-6 inhibitors, convalescent plasma

## Abstract

For the initial phase of pandemic of coronavirus disease 2019 (COVID-19), repurposing drugs that *in vitro* inhibit severe acute respiratory syndrome coronavirus-2 (SARS-CoV-2) have been attempted with overlooked or overestimated efficacy owing to limited clinical evidence. Most early clinical trials have the defects of study design, small sample size, non-randomized design, or different timings of treatment initiation. However, well-designed studies on asymptomatic or mild, or pediatric cases of COVID-19 are scarce and desperately needed to meet the clinical need. However, a trend could be observed based on current clinical evidence. Remdesivir and favipiravir may shorten the recovery time; lopinavir/ritonavir does not demonstrate treatment efficacy in severe patients. Triple therapy of ribavirin, lopinavir, and interferon β-1b showed early viral negative conversion, and the major effect may be related to interferon. Some small sample-size studies showed that interleukin-6 inhibitors may demonstrate clinical improvement; non-critical patients may benefit from convalescent plasma infusion in small sample-size studies; and the role of hydroxychloroquine or chloroquine in the treatment and prophylaxis of COVID-19 remains unclear. Combination therapy of traditional Chinese medicine with antiviral agents (ex. interferon, lopinavir, or arbidol) may alleviate inflammation in severe COVID-19 patients based on small sample-sized observational studies and experts’ opinion. Most of the published studies included severe or critical patients with COVID-19. Combination therapy of antiviral agents and immune-modulating drugs is reasonable especially for those critical COVID-19 patients with cytokine release syndrome. Drugs to blunt cytokine release might not benefit for patients in the early stage with mild disease or the late stage with critical illness. Traditional Chinese medicine with antiviral effects on SARS-CoV-2 and immune-modulation is widely used for COVID-19 patients in China, and is worthy of further studies. In this review, we aim to highlight the available therapeutic options for COVID-19 based on current clinical evidence and encourage clinical trials specific for children and for patients with mild disease or at the early stage of COVID-19.

## Introduction

Severe acute respiratory syndrome coronavirus-2 (SARS-CoV-2) is a fresh linage of β-coronavirus, which is closely to bat SARS-related coronavirus genetically (Chakraborty et al., 2020a). Since the outbreak of severe acute respiratory syndrome coronavirus-2 (SARS-CoV-2) in China toward the end of 2019, 32,110,656 cases were reported worldwide, and the virus caused 980,031 deaths, accounting for 3.05% of the infected population as of September 25, 2020 (World Health Organization, 2020). To date, no effective vaccines and promising antiviral agents for SARS-CoV-2 have been developed, and the currently available drugs are still under investigation. Therefore, many critically ill patients are treated with off-label antiviral drugs. Many drugs in emergency use are based on *in vitro* evidence or expert opinions. The clinical spectrum of COVID-19 ranges from asymptomatic carrier status or mild respiratory disease to severe pneumonia (Lai et al., 2020). COVID-19 tends to affect older patients with underlying medical conditions severely (Lai et al., 2020). In critical patients, lymphopenia, exhausted lymphocytes, and immune system activation due to concurrent cytokine release syndrome result in severe tissue damage (Cao, 2020b). Medications modulating inflammation may play a therapeutic role at this stage of disease. The publication of studies on COVID-19 has been fast tracked. Notably, two articles on the use of hydroxychloroquine or chloroquine for COVID-19 in the New England Journal Medicine and Lancet were retracted owing to suspicious data sources (Mehra et al., 2020a; Mehra et al., 2020b).

As no specific therapy is available for SARS-CoV-2, the proposed therapy is based on the previous experience from SARS or Middle East Respiratory Syndrome (MERS) coronavirus. These therapeutic molecules, for example viral methyltransferase inhibitors, nitazoxanide, protease inhibitors (such as lopinavir/ritonavir), interferon, therapeutic peptides, RNA synthesis inhibitors (such as ribavirin, flavirapivir, and remdesivir), anti-inflammatory drugs, and traditional Chinese drugs are not designed specifically against SARS-CoV-2 (Chakraborty et al., 2020c), and some of them are preclinical drugs, not used in humans yet.

**FIGURE 1:**
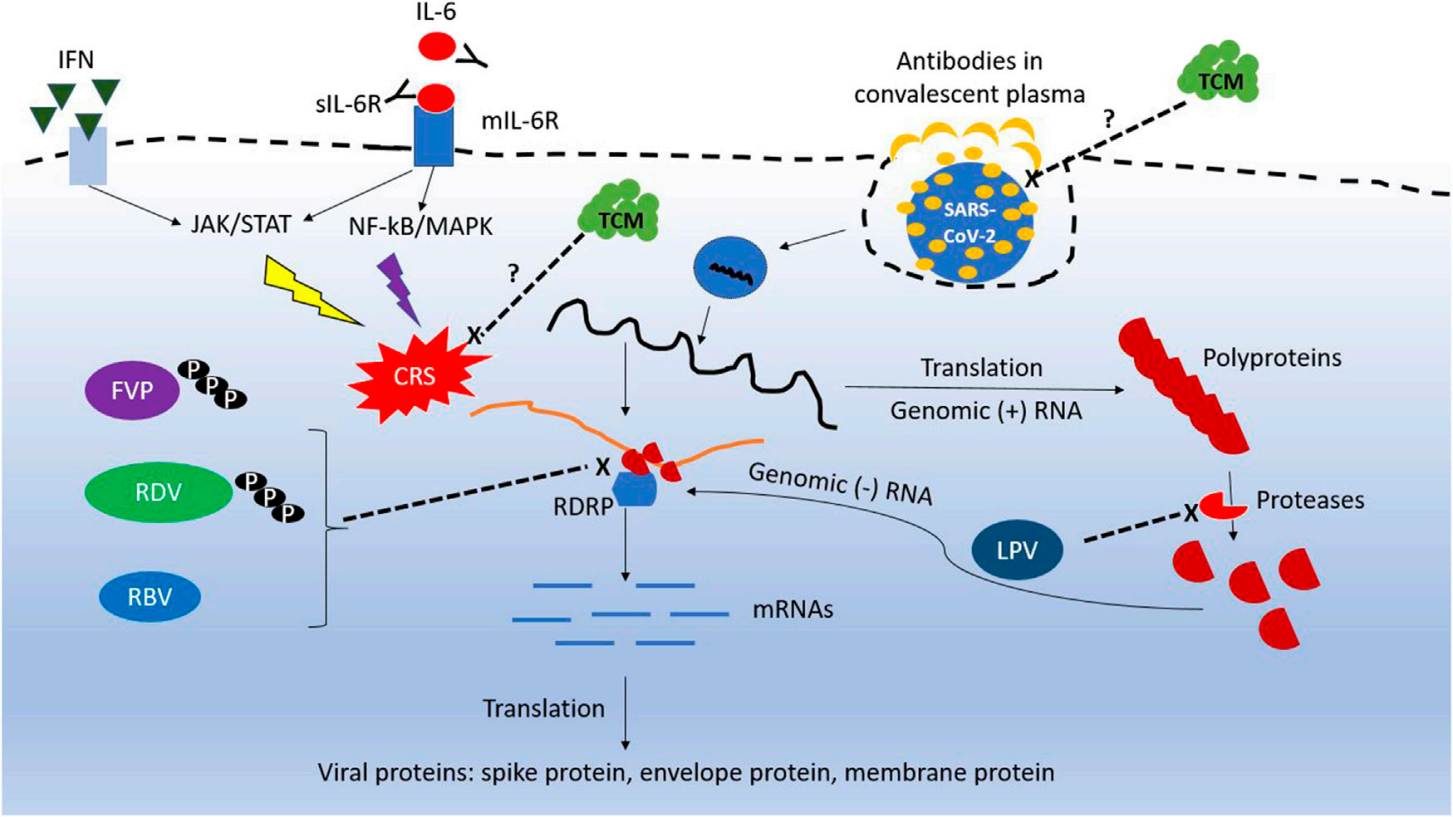
Conceptual diagram of the mechanism for repurposing antiviral agents against SARS-CoV-2. RDV, remdesivir; FVP, favipiravir; RBV, ribavirin; IFN, interferon; sIL-6R, soluble IL-6 receptor; mIL-6R, membrane IL-6 receptor; TCM, traditional Chinese medicine.

Most drugs currently used for COVID-19 are approved antiviral agents or antibodies against diseases other than COVID-19. The conceptual antiviral mechanisms for these drugs are summarized in Figure 1. Supporting evidence from well-designed clinical trials are limited, and there is a knowledge gap between clinical settings and sound scientific evidence. It is too early to conclude which drugs are effective against SARS-CoV-2, because most studies were conducted urgently with limited number of patients with severe disease. For example, several trials on convalescent plasma (CP) include those with limited cases, non-randomized-controlled design, and targeting patients who needed extracorporeal membrane oxygenation support.

Theoretically, therapy with effective antiviral agents in the early phase of COVID-19 may gain greater benefits. Agents with anti-inflammatory characteristics may benefit critical COVID-19 patients who have features of cytokine release syndrome in addition to administration of antiviral drugs. Therefore, both disease severity and timing of therapy initiation would affect the final clinical or virological outcomes.

In the early period of COVID-19 outbreak, although most patients had moderate or severe COVID-19, they received experimental drugs only when their clinical condition deteriorated. Clinical data of treatment in asymptomatic cases or patients with mild COVID-19 are lacking. Although children were believed to be less susceptible to SAR-CoV-2, critical COVID-19 diseases have been reported in children (Dong et al., 2020). Further studies to fill the knowledge gap regarding therapy for severe COVID-19 in children are warranted.

Before effective antiviral drugs for COVID-19 are available, current treatment options will come from repurposing drugs. Thus, in this review we aim to highlight potential therapeutic strategies from the viewpoints of clinicians based on updated clinical evidences, and provide a basis for future researches of effective antiviral therapies. In the future, the development of new drugs and vaccines relies on multidisciplinary cooperation among structural biologists, chemists, and medical doctors. A knowledge gap of pharmacotherapy for COVID-19 is expected to be filled up, if updated information can be shared among global research institutes (Chakraborty et al., 2020d).

## Remdesivir

Remdesivir, an RNA polymerase inhibitor, is a monophosphate prodrug that metabolizes to an active C-adenosine nucleoside triphosphate analog and shows activity against RNA viruses, such as Coronaviridae and Flaviviridae (Siegel et al., 2017). Triphosphate form of remdesivir is a substrate for RNA-dependent RNA polymerase complexes in coronaviruses and blocks viral RNA synthesis. Detailed mechanisms of remdesivir in inhibiting RNA polymerase is discussed in a review article by Saha et al. (2020a). It shows excellent *in vitro* activity against several coronaviruses, including SARS-CoV-2 with EC_50_ and EC_90_ values of 0.77 and 1.76 μM, respectively. Remdesivir is considered a potential therapy for COVID-19 in the beginning of outbreak (Al-Tawfiq et al., 2020; Wang M. et al., 2020). Holshue et al. reported the first case of COVID-19 treated with remdesivir in the United States; the patient’s condition improved one day after initiation of remdesivir (Holshue et al., 2020). However, it is unclear whether the use of remdesivir resulted in this improvement. After that, remdesivir was compassionately used in 53 cases, of which 68% showed improvement in oxygen support, 47% were discharged, and 13% died (Grein et al., 2020).

During February–March 2020, the first randomized, placebo-controlled trial of remdesivir in China showed no virological benefits or clinical effect in reducing the recovery time and deaths compared with the placebo group. Moreover, it caused several adverse effects leading to early termination of the trial (Wang Y. et al., 2020). Other clinical trials of remdesivir are ongoing; preliminary data from an international multicenter, placebo-controlled double-blind randomized trial suggest that remdesivir is effective in reducing the recovery time from 15 to 11 days in hospitalized patients (Beigel et al., 2020). On April 29, 2020, based on the Adaptive COVID-19 Treatment Trial, the National Institute of Allergy and Infectious Diseases in the United States announced that remdesivir was better than placebo in reducing recovery time in hospitalized patients with advanced COVID-19 and lung involvement (National Institute of Allergy and Infectious Diseases, 2020). Currently, remdesivir is being tested as a specific treatment for COVID-19 and has been authorized for emergency use in people with severe symptoms in the United States (Food and Drug Administration, 2020c).

The dosage regimen of remdesivir under investigation is a single 200 mg loading dose, followed by 100 mg daily infusion. Remdesivir demonstrated linear pharmacokinetics within this dose range and an intracellular half-life of more than 35 h (Sanders et al., 2020). Dose adjustment for patients with impaired hepatic or kidney function is not recommended. However, remdesivir therapy is not recommended in patients with an estimated glomerular filtration rate less than 30 ml/min (Sanders et al., 2020). Adverse effects, including gastrointestinal and hepatic (elevated transaminase levels) dysfunction, and infusion site reactions were reported to be associated with remdesivir therapy (Grein et al., 2020; Mehta et al., 2020). In a study without placebo, prolonged courses of 10-days remdesivir therapy did not show a significant difference from 5-days courses in patients with severe COVID-19 (oxygen saturation ≤94% in ambient air) not requiring mechanical ventilation (Goldman et al., 2020).

## Favipiravir

Favipiravir, an RNA polymerase inhibitor, is a prodrug of a purine nucleotide that inhibits viral replication (Sanders et al., 2020), and it demonstrated activity against SARSCoV-2 with a high EC_50_ of 61.88 μM/L (Wang M. et al., 2020). Various dosing regimens have been proposed based on the type of infection indication (Sanders et al., 2020); a loading dose of 2,400–3,000 mg every 12 h (two doses) has been considered for the treatment of COVID-19, followed by a maintenance dose of 1,200–1800 mg every 12 h (Mentre et al., 2015; Sanders et al., 2020). Favipiravir demonstrates a tolerable safety profile in terms of total and serious adverse effects compared with other drugs used for short-term treatment (Pepperrell et al., 2020). Adverse effects of favipiravir, namely hyperuricemia, teratogenicity, and QTc prolongation have not been adequately studied despite its long-term and widespread use against COVID-19 (Pepperrell et al., 2020). However, there is limited clinical experience with favipiravir for COVID-19 treatment (Li J. et al., 2020; Sanders et al., 2020).

In February 2020, the use of favipiravir in the experimental treatment of COVID-19 was investigated in China. In a prospective, randomized, multicenter study, favipiravir (1,600 mg every 12 h/first day followed by 600 mg every 12 h/day, n = 120) was compared with umifenovir (200 mg every 8 h/day, n = 120) for the treatment of moderate and severe COVID-19 infections for 10 days (Chen et al., 2020). On day 7, differences in clinical recovery were observed in patients with moderate infections (71.4% in favipiravir arm and 55.9% in umifenovir arm, *p* = 0.019). Favipiravir significantly improved the resolution time of pyrexia and cough. However, there were no significant differences between patients with mild/moderate disease and severe/critical disease in the subgroup analysis. The other trials are planned in over 40 countries (Consumer News and Business Channel, 2020).

## Lopinavir/Ritonavir

Lopinavir, an inhibitor of aspartate protease of human immunodeficiency virus (HIV), has been used in the treatment of HIV infection for a long time. Ritonavir can increase the concentration of lopinavir by inhibiting cytochrome P450. Lopinavir inhibits the action of protease 3CL^pro^ in HIV through C2-symmetric pocket, which is absent in coronavirus (Li and De Clercq 2020; Sheahan et al., 2020). Therefore, the inhibitory effect of lopinavir on SARS-CoV-2 is uncertain. In early May 2020, there was only one randomized controlled trial of lopinavir–ritonavir (LPVr), which was conducted in Wuhan, China (Cao et al., 2020a). In this study, 199 patients with COVID-19 pneumonia with oxygen saturation ≤94% in ambient air were included and randomly received LPVr 400/100 mg or standard care. As no differences were observed in the time to clinical improvement, 28-days mortality rate, and detectable viral load at various time points between LPVr therapy and standard care, many clinicians disapproved LPVr. However, some experts showed interest in LPVr owing to its benefits in lowering overall mortality and reducing the risk of respiratory failure or acute respiratory distress syndrome (Dalerba et al., 2020; Kunz, 2020). Furthermore, this study included patients with COVID-19 at a median of 13 days after disease onset. Antiviral agents may not be effective, because hyper-inflammatory response rather than viral burden may be responsible for critical COVID-19 (Kunz 2020). They advocated that LPVr combination should be retained in the therapeutic guideline until ongoing well-controlled, randomized trials are completed. The other clinical trials had methodological defects such as small sample size and unblinding after review (Doward and Gbinigie, 2020). LPVr cannot be considered beneficial for patients with COVID-19 in terms of primary outcome (Doward and Gbinigie, 2020). As LPVr is a safe and widely available drug and the present data do not completely exclude its therapeutic role in the early onset of COVID-19 (for example, LPVr is prescribed for moderate cases within 7–10 days after symptom onset), further trials designed for such clinical settings are warranted.

### Ribavirin and Interferon

Ribavirin, a guanosine analog which has distinct antiviral mechanisms, including both indirect (inosine monophosphate dehydrogenase inhibition and immunomodulatory effects) and direct mechanisms (interference with RNA capping, polymerase inhibition, and lethal mutagenesis) (Graci and Cameron, 2006). *In vitro* efficacy of ribavirin against SARS-CoV-2 viral strain WIV04 has been reported (Wang M. et al., 2020). In a single-center retrospective study conducted in Wuhan, China from January to February 2020, a total of 134 adults with severe COVID-19 were enrolled for analysis (Tong et al., 2020). Ribavirin therapy neither shortened the negative viral conversion time nor improved mortality rate. However, in a multicenter, prospective study conducted in Hong Kong, 127 patients were randomly assigned to the combination group (lopinavir 400 mg and ritonavir 100 mg every 12 h, ribavirin 400 mg every 12 h, and three doses of eight million international units of interferon β-1b on alternate days for 14 days) or control group (lopinavir 400 mg and ritonavir 100 mg every 12 h for 14 days) at a ratio of 2:1 (Hung et al., 2020). The median number of days from symptom onset to the start of study treatment was 5 days. There was no difference of adverse events between the two groups. The results showed that the combination group had a significantly shorter median time from the initiation of combination treatment to negative nasopharyngeal swab (7 days; interquartile range [IQR] 5–11 days) than the control group (12 days; IQR 8–15 days; hazard ratio 4·37; 95% confident interval [CI] 1·86–10·24]; *p* = 0·001). This study suggested early prescription of combination antiviral therapy may shorten the duration of viral shedding and improve symptoms in patients with mild to moderate COVID-19 (Hung et al., 2020). However, clinical efficacy of such a combination regimen for severe COVID-19 remains obscure. Theoretically adverse effects of ribavirin, such as anemia, hypomagnesemia, and bradycardia, should always be reminded (Muller et al., 2007). However, neither ribavirin monotherapy nor concurrent use with other medications (i.e. interferon or lopinavir-ritonavir) for the patients with COVID-19 did not increase the incidence of the former adverse effects, in comparison with the controls in previous studies (Hung et al., 2020; Tong et al., 2020). To sum up, clinical efficacy and safety of ribavirin warrant more clinical investigations.

As interferons are important cytokines which may be involved in eliminating virus-infected cells (Samuel, 2001), the combination of interferon along with other antiviral agents would be a logic strategy. Interferon β-1b appears to be a key player of combination therapy based on the published data (Hung et al., 2020). Therefore, future trials with interferon-based combination regimens, including different doses, types of interferon, or concomitant drugs, are worthy to be conducted.

## Chloroquine and Hydroxychloroquine

Chloroquine has been used clinically for more than 70 years. It is an approved anti-malarial drug; it is also used for autoimmune diseases. *In vitro* studies showed that chloroquine was highly effective in controlling SARS-CoV-2 infection of host cells at the entry and post-entry stages (Wang M. et al., 2020). The antiviral mechanisms of chloroquine are multifaceted. It can prevent nanoparticle uptake by macrophages via inhibiting the expression of phosphatidylinositol-binding clathrin assembly protein and subsequent clathrin-mediated endocytosis. In addition, chloroquine can prevent acidification of lysosomes, thereby inhibiting their fusion with endocytic vesicles (Hu et al., 2020).

A small open-label non-randomized trial conducted in France included 36 patients with COVID-19 (Gautret et al., 2020). The viral load was significantly lower in 20 cases treated with hydroxychloroquine sulfate 200 mg three times a day for 10 days than in those without treatment on day 6 post inclusion (70.0% vs. 12.5%, *p* = 0.0001). Notably, six cases treated with hydroxychloroquine (500 mg on day 1, followed by 250 mg per day for the next 4 days) and azithromycin achieved 100% viral clearance rate on day 6 (Gautret et al., 2020). In this study, six patients withdrew from the treatment arm. The study endpoint was the viral load of throat swabs on day 6 after treatment initiation, which was not a clinical outcome. However, poor study method and reporting were the drawbacks of the study (Ferner and Aronson, 2020). Thus, the routine use of these drugs for COVID-19 was recently regarded as premature and potentially harmful owing to side effects such as fatal arrhythmia, drug eruption, and hepatitis. A study including 84 patients with COVID-19 receiving oral hydroxychloroquine (given at dose of 400 mg twice on day 1, followed by 200 mg twice daily for 5 days) and azithromycin (given at daily dose of 500 mg for 5 days) was conducted in New York (Chorin et al., 2020). The included patients were followed up with electrocardiography (ECG) on an average of 4.3 ± 1.7 days. The results demonstrated prolongation of QTc from a baseline average of 435 ± 24 ms to a maximal average value of 463 ± 32 ms (*p* < 0.001). Notably, 11% of patients experienced severely prolonged QTc (>500 ms), which may cause arrhythmia and sudden cardiac death. Therefore, hydroxychloroquine should be used with caution, particularly in patients with co-morbidities and those who take QT-prolonging drugs. QTc assessment by ECG at the beginning and after therapy is warranted. The United States Food and Drug Administration (FDA) also cautions against the use of these drugs for COVID-19 outside the hospital setting or in a clinical trial owing to the risk of heart rhythm problems (Food and Drug Administration, 2020a).

## Interleukin-6 Inhibitors

Some patients with COVID-19 develop considerable inflammation associated with multiorgan failure requiring intensive care, and their severity and mortality of COVID-19 is associated with high levels of serum cytokines. Of note, high levels of pro-inflammatory cytokine and IL-6 were noted in severe COVID-19 patients (Giamarellos-Bourboulis et al., 2020; Qin et al., 2020; Wang C. et al., 2020). Such cytokine release syndrome was initiated through JAK-STAT or MAPK/NF-κB-IL-6 pathway. Tocilizumab, a humanized monoclonal antibody, is able to bind both membrane bound receptors and soluble receptors for IL-6, and a potential drug for patients with severe COVID-19 (Saha et al., 2020b). Tocilizumab (TCZ), a humanized antibody targeting IL-6 receptor (IL-6R), has been licensed for the treatment of rheumatoid arthritis, juvenile idiopathic arthritis, giant cell arteritis, and chimeric antigen receptor T-cell therapy-induced cytokine release syndrome (Rubbert-Roth et al., 2018). Sarilumab, another IL-6R inhibitor for rheumatoid arthritis, and siltuximab, a chimeric monoclonal antibody against IL-6, are indicated for multicentric Castleman’s disease (Boyce et al., 2018; van Rhee et al., 2018).

Several retrospective, non-randomized clinical studies reported favorable outcomes in patients treated with TCZ. A case-control study including 20 patients with COVID-19 receiving TCZ and 25 control patients reported fewer deaths and/or ICU admissions in the TCZ group (25% vs. 72%, *p* = 0.002) (Klopfenstein et al., 2020). TCZ has been associated with a lower risk of death in a before–after study of 85 non-intubated patients with COVID-19 having respiratory distress [hazard ratio of death: 0.035 (0.004–0.347, *p* = 0.004)] (Capra et al., 2020). Another cohort study including 239 patients reported a higher than expected survival rate in severe patients treated with TCZ (Price et al., 2020). However, a retrospective cohort study including 65 patients (32 receiving TCZ) showed no significant difference in 28-days mortality (15% vs. 33%, *p* = 0.15) (Campochiaro et al., 2020). In summary, many case series demonstrated clinical improvement, radiological resolution, and decreased inflammation markers (Morena et al., 2020; Sciascia et al., 2020; Toniati et al., 2020; Xu et al., 2020). TCZ dosing in the reported studies ranged from 80 to 700 mg per dose, usually 4–8 mg/kg, in one or two doses. As TCZ is an immunosuppressant, its administration is occasionally associated with elevated liver enzymes, thrombocytopenia, and neutropenia (Morena et al., 2020). A substantial risk of bacteremia was reported when TCZ was administered in combination with methylprednisolone at a dose of 1 mg/kg/day (Giacobbe et al., 2020).

Other IL-6 inhibitors, such as siltuximab and sarilumab, have been reported as salvage therapy for COVID-19. Siltuximab 11 mg/kg (one or two doses) was administered in 21 patients with COVID-19 receiving either continuous positive airway pressure therapy or non-invasive ventilation, and 76% showed clinical improvement or stable condition (Gritti et al., 2020). Sarilumab at a dose of 400 mg, followed by 200 mg twice in the subsequent days was administered in eight patients, and seven of them survived to discharge (Benucci et al., 2020).

As the current evidence supporting the use of IL-6 inhibitors for treating COVID-19 is weak, professional societies do not recommend its use outside clinical studies (Alhazzani et al., 2020; Bhimraj et al., 2020). Dozens of randomized, placebo-controlled trials of IL-6 inhibitors, such as clazakizumab, tociluzmab, sarilumab, or olokizumab, are ongoing (ClinicalTrials.gov, 2020), and were summarized and updated in Table 1 till September 09, 2020. Research outcomes for these studies are expected to fill the knowledge gap in the treatment efficacy of IL-6 inhibitors for patients with COVID-19.

**TABLE 1:** Summary of current randomized, placebo-controlled clinical trials of IL-6 inhibitors for COVID-19.

Drugs	Indication	Interventions	Multicenter	No. of cases	Phase of trail	Country	Registered no
Clazakizumab	Life-threatening COVID-19 infection	Clazakizumab vs. placebo	No	30	II	United States	NCT04381052
Clazakizumab	Life-threatening COVID-19 infection	Clazakizumab 25 vs. 12.5 mg vs. placebo	No	90	II	United States	NCT04343989
Clazakizumab	Life-threatening COVID-19 infection	Clazakizumab vs. placebo	No	30	II	United States	NCT04363502
Tocilizumab	COVID-19 pneumonia	Tocilizumab vs. placebo	Yes	379	III	United States	NCT04372186
Tocilizumab	Moderate to severe COVID-19 pneumonia	Tocilizumab vs. placebo	Yes	450	III	United States	NCT04320615
Tocilizumab	Severe COVID-19 pneumonia	Remdesivir + tocilizumab vs. Remdesivir + placebo	Yes	450	III	United States	NCT04409262
Tocilizumab	COVID-19 disease	Tocilizumab vs. placebo	Yes	100	II	Switzerland	NCT04335071
Tocilizumab	SARS-CoV-2 infection and evidence of systemic inflammation	Tocilizumab vs. placebo	Yes	243	III	United States	NCT04356937
Sarilumab	Hospitalized COVID-19 patients	Sarilumab vs. placebo	Yes	1912	II//III	United States	NCT04315298
Sarilumab	Severe or critical COVID-19	Sarilumab dose 1 and dose 2 vs. placebo	Yes	409	III	Argentina	NCT04327388
Olokizumab	Severe COVID-19	RPH-104^a^ vs. Olokizumab vs. placebo	Yes	372	III	Russia	NCT04380519

Data resource: *ClinicalTials.gov*, accessed on 9 September 2020.

a RPH-104, a novel heterodimeric fusion protein, capable of inhibition of human IL-1 beta/IL-1F2 signaling pathway.

## Convalescent Plasma

Passive antibody administration for infectious diseases was introduced in the 1890s and has been largely replaced by antimicrobial agents in the 20th century (Casadevall and Scharff, 1995). CP became a treatment option for severe viral diseases such as SARS, Middle East respiratory syndrome, influenza A H1N1/2009, and Ebola virus disease with variable results, because no specific treatment was available for these diseases (Cheng et al., 2005; Yeh et al., 2005; Hung et al., 2011; Mair-Jenkins et al., 2015; van Griensven et al., 2016).

Many physicians hope that CP transfusion would be effective in treating COVID-19. In addition, the FDA (United States) accepted applications for expanded access and single patient emergency use of CP (Food and Drug Administration, 2020b). Up to 33% of patients who recovered from COVID-19 generated very low titers of SARS-CoV-2 neutralizing antibodies. Therefore, neutralizing antibody testing is highly recommended for CP donors (Robbiani et al., 2020; Wu et al., 2020).

A prematurely terminated, possibly underpowered, randomized controlled trial involving 103 patients with severe or life-threatening COVID-19 who received CP at a dose of 4–13 ml/kg showed no significant difference in the rate of clinical improvement within 28 days (51.9% vs. 43.1%, difference 8.8% [95% CI: −10.4–28.0%]). In the subgroup analysis, only patients with life-threatening disease were more likely to improve after CP transfusion (hazard ratio, 2.15; 95% CI, 1.07–4.32). The 72-h negative conversion rate of viral nucleic acid detection was higher in the CP group (87.2% vs. 37.5%, *p* < 0.001) (Li L. et al., 2020).

Seven case series reported the treatment outcomes of CP in patients with COVID-19 with disease severity ranging from mild disease to severe respiratory failure on extracorporeal membrane oxygenation (Ahn et al., 2020; Duan et al., 2020; Pei et al., 2020; Salazar et al., 2020; Shen et al., 2020; Ye et al., 2020; Zhang B. et al., 2020). In these case series, clinical improvement, radiological resolution of pneumonia, and decreased viral load were observed in most patients; however, studies with comparable groups showed less promising results. One study reported a high fatality rate in both groups (5/6 in the CP group and 14/15 in the control group), and another study showed only slight improvement in oxygen and laboratory values (Hegerova et al., 2020; Zeng et al., 2020). The volume of CP in the above studies ranged from 200 to 600 ml per time for one or two sessions. The safety of CP transfusion was supported by the initial analysis of 5,000 patients involved in the US FDA Expanded Access Program, which demonstrated serious adverse events in 0.5% of CP recipients (Joyner et al., 2020). More than 10 randomized controlled trials have been commenced to clarify the efficacy of CP in the treatment of COVID-19 (ClinicalTrials.gov, 2020).

## Traditional Chinese Medicine

In the early outbreak in China, no drugs approved for the treatment of COVID-19 were limited. Therefore, the China official guideline suggested traditional Chinese medicine (TCM) in combination with antiviral drugs for COVID-19 patients (Novel Coronavirus Pneumonia Diagnosis and Treatment Plan, Provisional 7th ed., 2020). In China, TCM wards were set up in the hospitals and some COVID-19 cases were treated accordingly. Of note, Qingfei Paidu Decoction (QPD) has been strongly recommended for confirmed cases in different categories in the official guidelines based on the practical clinical experiences by the China official guideline. QPD comprises 21 traditional Chinese medicines, which are expected to have protective effects for different organs, in addition to lung. Of the components in QPD, five are supposed to possess *in vitro* suppressive effects on SARS virus (Yu et al., 2012; Zhang D. H. et al., 2020).

In a retrospective, one-center study conducted in Hubei Province, China between January and February 2020, 63 patients with COVID-19 treated with antiviral agents alone (n = 26) and in combination with QPD (n = 37) were analyzed (Xin et al., 2020). Antiviral agents used in this study included interferon, arbidol, or lopinavir. Before QPD treatment, the combination group had higher blood levels of C-reactive protein (CRP) and more pulmonary inflammation and clinical symptoms than the antiviral group. At the end of treatment, death rate, length of hospital stay, and improvement of pulmonary CT score were similar in both groups. Of note, CRP, creatine kinase, creatine kinase-myocardial band, lactate dehydrogenase, and blood urea nitrogen levels were improved only in the QPD therapy group (all *p* < 0.05). Although QPD has both antiviral and anti-inflammatory potential, its precise treatment effect for COVID-19 needs to be clarified and therapeutic efficacy requires clinical validation through well-designed randomized-control studies.

### Vaccines in Development

In addition to antiviral agents, the global use of COVID-19 vaccine is a promising strategy to end the current pandemic. Dozens of COVID-19 vaccines designed by different organizations are at different phases of clinical trials. Saha *et al.* summarized a total of 146 COVID-19 vaccines, including live-attenuated vaccine, inactivated or killed vaccine, subunit vaccine, and nucleic acid-based vaccine, engaged in clinical trials (Saha et al., 2020c), and the number of candidates will be increasing in the future. With the aid of immunoinformatics, scientists could select suitable peptide sequences which are potential B- or T-cell epitopes for the generation of epitopic vaccines against SARS-CoV-2 (Bhattacharya et al., 2020a; Bhattacharya et al., 2020b). Another example is the peptide vaccine against spike glycoprotein with molecular docking on toll-like receptor-5 (TLR5), which can evoke early innate immune response against COVID-19 (Chakraborty et al., 2020b). However, the efficacy of these candidate vaccines remains to be verified.

## Conclusion

The COVID-19 pandemic is still severe, and most of the drugs currently available for COVID-19 are not designed specifically against SARS-CoV-2. The search for effective antiviral agents specific to SARS-CoV-2 is still ongoing. The potential drugs for COVID-19 are summarized in Table 2. Ideally, the inhibition of viral proliferation in early stage of COVID-19 can prevent subsequent severe complications. In contrast, critical cases of COVID-19 benefit from anti-inflammation therapy in conjunction with antiviral agents, as in late stage cytokine release syndrome is the main cause of multi-organ failure and even death. Clinical evidence suggests that remdesivir can shorten the recovery time of advanced COVID-19 pneumonia. However, the clinical efficacy and safety of other agents for emergency use is controversial owing to the limitations of study designs. IL-6 inhibitors, which alleviate severe inflammation induced by cytokine release after viral infection, may improve clinical outcome of critical cases of COVID-19. Several clinical trials of IL-6 inhibitors for severe COVID-19 patients are conducted. It is still too early to draw conclusions until more evidences from well-designed clinical trials are available. Traditional Chinese herbs are worthy of further investigations. However, the precise antiviral mechanisms of traditional Chinese medicines remain difficult to be explored, as the regimens often contain multiple components. In addition, their therapeutic efficacy needs concrete evaluations based on well-designed clinical studies. Although supporting evidences for other antiviral agents and convalescent plasma are less than those for remdesivir, the clinical application of the aforementioned therapies may still be considered in the real world setting for patients with severe COVID-19, if no contraindications are present, owing to the limited treatment choices.

**TABLE 2:** Repurposing of potential candidate drugs for COVID-19.

Therapy/Mechanism	Authors	Country	Phase of trails	Methods	Primary outcome	Report findings
*Remdesivir* (RDV)/RNA polymerase inhibitor	Wang *et al*	China	III	Double-blinded RCT; RDV (n = 158) vs. placebo (n = 79)	Clinical improvement up to 28 days	RDV was not associated with a difference in time to clinical improvement (hazard ratio 1.23 [95% CI 0.87–1.75])
	Beigel *et al*	United States	III	Double-blinded; RDV (n = 538) vs. placebo (n = 521); hospitalized patients; favorable outcome in preliminary report	Time to recovery	Median recovery time: 11 days vs. 15 days, recovery rate ratio, 1.32; 95% CI, 1.12–1.55; *p* < 0.001
*Lopinavir-ritonavir* (LPV/r)/protease inhibitor	Cao *et al*	China	N/A	Hospitalized patients with COVID-19 and respiratory illness^a^; open-label RCT; LPV/r (n = 99) vs. placebo (n = 100)	Time to clinical improvement	Modified intention-to-treat population^b^: Median time to clinical improvement: 15 days vs. 16 days (hazard ratio, 1.39; 95% CI, 1.00–1.91)
*Favipiravir* (FPV)/RNA polymerase inhibitor	Chen *et al*	China	N/A	Adults with COVID-19 pneumonia; open-label RCT; FPV (n = 120, 116 accessed) vs. UFV (n = 120)	Clinical recovery rate on day 7	Clinical recovery rate on day 7: 71.4% vs. 55.9%; rate ratio (95% CI): 0.16 (0.03–0.28)
*Ribavirin* (RBV)/Guanosine analog	Tong *et al*	China	N/A	Adults with severe COVID-19; retrospective study; RBV (n = 44) vs. none (n = 71)	Time to viral negative conversion	12.8 ± 4.1 vs. 14.1 ± 3.5 days (*p* = 0.314)
*Interferon* (*IFN*) *β-1b, RBV, LPV/r/*Combination therapy	Hung *et al*	Hong Kong	II	Adults with admitted COVID-19 patients; open-label RCT	Time to viral negative conversion	7 days vs. 12 days (hazard ratio,4.37; 95% CI (1.85 ∼ 10.24), *p* = 0.0010)
*Chloroquine* (CQ) and *hydroxychloroquine* (HCQ)	Gautret *et al*	France	III	Hospitalized patients with COVID-19 (age >12 years) regardless of their clinical status; open-label, non-RCT; HCQ ± AZI (n = 26) vs. none (n = 16)	Viral clearance at day 6	70.0% vs. 12.5%, *p* = 0.001
	Mehra *et al*	Multinational	N/A	Hospitalized patients with COVID-19 received treatment of interest within 48 h; multinational registry analysis, CQ or HCQ ± macrolide (n = 14,888) vs. none (n = 81,144)	In-hospital mortality and *de novo* ventricular arrhythmias	Retracted owing to suspicious data sources
*Tocilizumab* (TCZ)/Interleukin-6 inhibitors	Klopfenstein *et al*	France	II	Hospitalized adult patients with COVID-19; TCZ (n = 20) vs. control (n = 25)	Death and/or ICU admission	Patients with TCZ presented with severe form; death and/or ICU admission: TCZ 25% vs. control 72%, *p* = 0.002
	Campochiaro *et al*	Italy	II	Severe non-ICU adult patients with COVID-19; TCZ (n = 32) vs. control (n = 33)	Survival and clinical improvement at 28 days	Clinical improvement: TCZ 69% vs. control 61%, *p* = 0.61; mortality rate: TCZ 15% vs. control 33%, *p* = 0.15
	Price *et al*	United States	II	Hospitalized patients with COVID-19; observational study; TCZ (n = 153) vs. control (n = 86)	—	TCZ-treated patients with similar survival rates to non-severe patients (83% vs. 91%, *p* = 0.11)
*Convalescent plasma* (CP)/Neutralizing antibodies	Li *et al*	China	II	Severe and life-threatening COVID-19; RCT; CP (n = 52) vs. standard treatment (n = 51)	Clinical improvement within 28 days	28-days clinical improvement: CP 51.9% vs. standard treatment 43.1%; hazard ratio, 1.40 (95% CI, 0.79–2.49), *p* = 0.26. Patients without life-threatening disease: 28-days clinical improvement, hazard ratio, 2.15 (95% CI, 1.07–4.32); 72-h negative conversion rate of viral nucleic acid detection was higher in the CP group (87.2% vs. 37.5%, *p* < 0.001)
	Hegerova *et al*	United States	II	Adult patients with severe or critical COVID-19; case-control; CP (n = 20) vs. matched control (n = 20)	Clinical outcomes up to 14 days	Mortality rate: CP 10% vs. control 30%, *p* = 0.11; mechanical ventilation: CP 30% vs. control 5%, *p* = 0.1
	Zeng *et al*	China	II	Adult patients with COVID-19 and respiratory failure; case series, observational; CP (n = 6) vs. control (n = 15)	Primary outcome: Fatality and secondary outcome: Virus shedding	Mortality rate: CP 83.3% vs. control 93.3%; duration of virus shedding: CP 23.5 days vs. control 20.0 days, *p* = 0.38
*Qingfei paidu qecoction* (QPD)/Traditional Chinese medicine	Xin *et al*	China	II	COVID-19 patients admitted to the hospital (>15 years old); retrospective study; QPD + western medicines (interferon, lopinavir, or arbidol; n = 37) vs. Western medicine (n = 26)	Improvement of inflammatory markers at the end of treatment	% Of normal CRP,TLC, LDH level: Combined group > control group (*p* < 0.05)

AZI, Azithromycin; RCT, randomized-controlled trial; UFV, umifenovir; CRP, C-reaactive protein; TLC, total lymphocyte count; LDH, lactate dehydrogenase; N/A, not applicable.

a Oxygen saturation ≤94% in room air or arterial oxygen partial pressure/fractional inspired oxygen (PaO2/FiO2) ≤300 mmHg.

b Exclude three early deaths.

In summary, COVID-19 vaccine is the most promising strategy to end the current pandemic in addition to anti-viral agents. Design of novel anti-viral agents which are specific for SARS-CoV-2 will provide more effective therapy for COVID-19 patients. Development of effective vaccines and anti-viral drugs both needs multidisciplinary cooperation. Before effective vaccines and anti-viral drugs are available, therapy with repurposing drugs are still the mainstream. Drugs which suppress virus may benefit patients in the early phase of COVID-19. Agents with anti-inflammatory characteristics may benefit critical COVID-19 patients who have features of cytokine release syndrome in addition to administration of antiviral drugs. Traditional Chinese medicines may have advantages in aspects of immunomodulation and suppressing virus when administrating with other anti-viral agents.

## Author Contributions

PLC, NYL, and CTC reviewed and analyzed the data. PCC, NYL, CTC, WCK and PRH prepared the manuscript. PLC, NYL, CTC, WCK and PRH read and approved the final version of the manuscript.

## Conflict of Interest

The authors declare that the research was conducted in the absence of any commercial or financial relationships that could be construed as a potential conflict of interest.
